# Structural Morphology and Optical Properties of Strontium-Doped Cobalt Aluminate Nanoparticles Synthesized by the Combustion Method

**DOI:** 10.3390/ma15228180

**Published:** 2022-11-17

**Authors:** Sivaraman Kanithan, Natarajan Arun Vignesh, Siva Baskar, Santhosh Nagaraja, Mohamed Abbas, Abdul Aabid, Muneer Baig

**Affiliations:** 1 Department of Electronics and Communication Engineering, MVJ College of Engineering, Whitefield, Bengaluru 560067, India; 2 Department of ECE, Gokaraju Rangaraju Institute of Engineering and Technology (GRIET), Hyderabad 500090, India; 3 Department of Humanities and Sciences, KG Reddy College of Engineering and Technology, Chilkur Village, Hyderabad 500075, India; 4 Department of Mechanical Engineering, MVJ College of Engineering, Whitefield, Bengaluru 560067, India; 5 Electrical Engineering Department, College of Engineering, King Khalid University, Abha 61421, Saudi Arabia; 6 Electronics and Communications Department, College of Engineering, Delta University for Science and Technology, Gamasa 35712, Egypt; 7 Department of Engineering Management, College of Engineering, Prince Sultan University, P.O. Box 66833, Riyadh 11586, Saudi Arabia

**Keywords:** CoAl_2_O_4_ nanoparticles, cubic spinel, optical properties, ferromagnetic materials

## Abstract

The study of structural morphology and the optical properties of nanoparticles produced by combustion methods are gaining significance due to their multifold applications. In this regard, in the present work, the strontium-doped cobalt aluminate nanoparticles were synthesized by utilizing Co_1−*x*_Sr*_x_*Al_2_O_4_ (0 ≤ *x* ≤ 0.5) L-Alanine as a fuel in an ignition cycle. Subsequently, several characterization studies viz., X-ray diffraction (XRD), energy-dispersive X-ray (EDX) analysis, high-resolution scanning electron microscopy (HRSEM), Fourier transform infrared spectroscopy (FTIR), ultraviolet (UV) spectroscopy and vibrating sample magnetometry (VSM) were accomplished to study the properties of the materials. The XRD analysis confirmed the cubic spinel structure, and the average crystallite size was found to be in the range of 14 to 20 nm using the Debye–Scherrer equation. High-resolution scanning electron microscopy was utilized to inspect the morphology of the Co_1−*x*_Sr*_x_*Al_2_O_4_ (0 ≤ *x* ≤ 0.5) nanoparticles. Further, EDS studies were accomplished to determine the chemical composition. Kubelka–Munk’s approach was used to determine the band gap, and the values were found to be in the range of 3.18–3.32 eV. The energy spectra for the nanoparticles were in the range of 560–1100 cm^−1^, which is due to the spinel structure of Sr-doped CoAl_2_O_4_ nanoparticles. The behavior plots of magnetic induction (M) against the magnetic (H) loops depict the ferromagnetic behavior of the nanomaterials synthesized.

## 1. Introduction

Nanoscience and nanotechnology have gained significance in various domains of electronics, material science, physics, chemistry, etc. In this regard, the research on the synthesis of functional materials and control of the nanoparticle’s size and length has a profound impact [1,2,3,4,5,6,7,8]. Hence, the synthesis of nanoparticles and related research found a greater impact, especially for the development of advanced systems and structures. Nanotechnology-adopted structural synthesis, exploration, material characterization, utilization, etc., have several practical applications, especially for the real-time synthesis of nanoelectromechanical systems. In this regard, fundamental studies on the synthesis of Co-based nanoparticles have great significance. Thus, Co_1−*x*_Sr*_x_*Al_2_O_4_ (0 ≤ *x* ≤ 0.5) nanoparticles have shown an expansive scope with astounding characteristics that make them suitable for numerous applications and whose qualities make them suitable for use in nanomedical devices, nanoelectromechanical systems, etc. [9,10,11,12,13,14]. The synthesis of nanoparticles is greatly impacted by fabrication strategies and the composition of the compounds [15,16,17,18,19,20,21,22]. The nano-base materials have been picked as a subject of examination in this work due to the commitment displayed in showing the non-linear optical properties of these materials for several combinations of dopants [23,24,25,26]. The present study deals with the preparation and characterization of Sr-doped CoAl_2_O_4_ nanoparticles. Additionally, various composites such as Zn, Mg, and Cu can be effectively used to replace the atoms of host Sr-doped CoAl_2_O_4_ nanoparticles, and the nanoparticles are synthesized by the combustion technique [27,28,29,30,31]. The dopant concentrations are limited to 25% to obtain pure-phase samples under different experimental conditions. These samples have been analyzed for their various experimental techniques. In particular, quantum confinement effects and the surface-to-volume ratio of nano-sized CoAl_2_O_4_ modify the photosensitive, magnetic, dielectric, and electronic properties compared to the properties of their bulk counterparts. Further, the nanospinels of CoAl_2_O_4_ at the nanoscale region are successfully utilized as pigment layers on luminescent materials and color filters for automotive lamps. The exchange of electrons from the beginning to a higher energy state is the establishment of the capability of the spectrophotometer. The electron spin in each sub-atomic orbital should be matched in the ground state; when they are matched in a higher energy state, the state is alluded to as an energized singlet state [32,33,34,35,36]. If the spin of the electrons is parallel in the excited state, then it is known as the excited triplet state [37,38,39,40,41]. Hence, it is important to understand the characteristics of the nanoparticles synthesized in order to evaluate their mechanical, electrical, magnetic, and thermo-electrical behavior. In this regard, the present work focuses on the synthesis and characterization of the structural and optical morphology of the strontium-doped cobalt aluminate nanoparticles.

## 2. Experimental Procedure

### 2.1. Synthesis

Sr^2+^ ion-doped CoAl_2_O_4_ spinel nanoparticles are synthesized using cobalt nitrate (Co(NO_3_)_2_·6H_2_O), aluminum nitrate (Al(NO_3_)_3_·9H_2_O), strontium nitrate (Sr (NO_3_)_2_), and L-alanine (C_3_H_7_NO_2_) compounds, which act as fuel for the combustion method. The reagent-grade compounds were procured from SD Fine, India. They were utilized without any additional purification.

The homogeneous solution of the compounds is prepared by mixing the strontium, cobalt, and aluminum nitrate in a 1:2 molar proportion. They are then added to the aluminum nitrate and L-alanine solution and stirred using an ultrasonic-assisted stirrer for one hour. Here, the nitrate is combined with an oxidizer with L-alanine as a fuel. This mixed solution is poured into a 50 mL Teflon beaker and placed in an autoclave. The autoclave is then inserted in a hot air oven at 160 °C for 12 h. Post-combustion, the compounds are slowly cooled down to room temperature. The final product is obtained in the form of precipitates and the precipitated particles are washed with ethanol and double-deionized water and dried at 100 °C. The ratio of oxidizer/fuel (F/O) charge considered in the present work is 1. For the combustion cycle, the homogeneous mixture is set in a silica cauldron and kept in a hot air oven for 120 minutes. The nanoparticles Co_1−*x*_Sr*_x_*Al_2_O_4_ (0 ≤ *x* ≤ 0.5) are synthesized through the chemical reaction in the hot air oven. Thereby, the nanoparticles obtained after the combustion are cleaned with distilled water and ethanol at room temperature. The final product was then labelled as CoAl, CoAS2, CoSA3, and CoSA4 based on the different molar ratios of strontium (0, 0.2, 0.3, and 0.4) added to the CoAl_2_O_4_ compounds. The chemical reaction involved in the formation of Co_1−*x*_Sr*_x_*Al_2_O_4_ (0 ≤ *x* ≤ 0.5) nanoparticles throughout the combustion process employing L-alanine as a precursor is given in Equation (1).

Co(NO_3_)_2_ 6 H_2_O _(S)_ + 2 Al(NO_3_)_3_ 9 H_2_O _(S)_ + 2.666 C_3_H_7_NO_2 (S)_ → CoAl_2_O_4 (S)_ + 10.66 N_2 (g)_ ↑ _+_ 8 CO_2 (g)_ ↑ + 33.33 H_2_O _(g)_ ↑ (1)

### 2.2. Characterization Studies

Cobalt aluminates are nanoparticles whose formation is ascertained by utilizing the X-ray diffractor at λ = 1.5406 Å radiation in the 2θ range of 20° to 80° (Model Rigaku Ultima III). A Malvern Panalytical Ltd. (United Kingdom), make Raman spectroscopy X-ray scanner connected with an EIKO IB2 electron scanner available at the Central Instrumentation Facility of IIT Madras, Chennai, India is used to conduct morphological and element testing [42,43,44,45,46,47,48]. The diffuse reflectance range is recorded utilizing a two-fold pillar spectrophotometer in the 300–700 nm region to decide the band holes. The FT-IR spectrum is recorded on a Perkin Elmer spectrophotometer (Spectrum RX1), available at the Central Instrumentation Facility of IIT Madras, Chennai, India. The phase confirmation and unit cell dimensions can be accomplished by powder X-ray diffraction (XRD) studies. The cathode-ray tube generates X-rays which are filtered to produce monochromatic radiation and are subsequently collimated [49,50,51,52,53,54]. The collimated beam is made to fall on the sample and because of this, constructive interference takes place. This happens due to the interaction of X-rays with the sample, thereby, it satisfies Bragg’s law (*n*λ = 2*d* sinθ). The distinct X-rays which are diffracted give an overview of the unit cell characteristics [55,56,57,58,59,60]. The samples are examined for the two-point scope of conceivable cross-section diffraction bearings that come out of the powdered material’s arbitrary direction [61,62,63,64,65]. Since each material under study has a unique set of d-spacings, the obtained diffraction peaks were converted to d-spacing, which is compared with the standard reference patterns [66,67]. Further, FTIR spectroscopy is carried out in the present work to measure the frequencies of radiation being absorbed by the molecules of the nanoparticles synthesized [68,69,70,71,72,73].

### 2.3. Ultraviolet Rays-Observable Spectroscopy

UV-observable spectroscopy is used in the present work to study the visual as well as the electronic property of nanomaterials which work on the basic principle of measuring the light absorption by a sample [74,75,76,77,78]. In general, approximately 200 nm to 800 nm is the frequency range that spectrometers cover. Additionally, a further extension of a measurement beyond 800 nm is possible, but it can be carried out using optics, a detector, or different light sources [79,80].

The ultraviolet spectrum is simple to compute and at the same time, beginners tend to make common mistakes. The first condition is that the concentration of the sample should not be too high. Absorbance (A) or a visual concentration and transmittance value should lie between 1 and 3. A higher sample or OD levels usually tend to saturate absorption and distort the spectrum. Second, before analyzing the sample spectrum, the background must be corrected. The same cuvette and solvent must be used for all characterizations. Third, before analyzing, the sample should be free of floaters in the solution; the floaters should be clear so that they are visible to human eyes, as the distortion of the spectrum occurs when there is significant scattering due to visible floaters. The fundamental source of spots in nanoparticle liquids is particle dispersion or coagulation. The particles are large structures, and hence, are clearly visible to the human eye via an electron microscope. The centrifugation process must be carried out before the analysis to filter out a small number of aggregates that are present in the sample.

### 2.4. HR-SEM Techniques

The surface morphology of any material under study can be obtained using HR-SEM images. In the case of a light microscope, visible light is used, whereas in the case of electron microscopes, electrons are used for imaging purposes, and hence, the resolution is better in the case of SEM. A specific set of coils was used by SEM to scan the beam in a raster-like pattern. Electrons interact with samples based on which signals are emitted, thus, providing information about the morphology chemical composition and crystalline nature of the sample.

To protect the instrument from contamination, vibrations, or noise and to obtain a high-resolution image, the source of the electron is wrapped and kept within a chamber to conserve a vacuum [81]. The electron path is regulated by magnetic lenses that are made of coils of wire contained inside metal pole pieces. The magnetic field is produced as the current flows through the coils [82,83]. There are two distinct types of electromagnetic lenses. Before the electron beam cone opens, the condenser lens converges the incoming beam first. Later, the objective lens converges the beam one more time before it interacts with the sample, whereas the objective lens causes the electron beam to focus on the sample [84,85,86,87,88]. The beam, using scanning coils, is on the right-hand side of the sample’s surface. When an electron interacts with the sample, it gives signals which contain secondary electrons which produce SEM images and provides information about the morphology and topography of the sample, crystal structure, and orientation of minerals obtained from backscattered electrons (BSE); in addition, characteristic X-rays are used to determine the presence of elements in the sample.

Characteristic X-rays are produced when inelastic collisions take place between incident electrons and beat discrete orbital (shells) electrons in atoms in the sample [89]. When the excited electrons come back to the ground state, X-rays of a fixed wavelength are obtained as various orbital (shell) for a given element.

### 2.5. EDAX Techniques

The presence of elements in a sample under study can be analyzed using EDAX analysis. When X-rays are emitted, the interaction between electromagnetic radiation and matter is analyzed. Atoms in the sample in a state of rest include quantum mechanical particles in distinct energy bands or orbitals that are bonded to the nuclei. The expelled inner shell particle’s location is filled by a particle from an external layer with much more energy; in addition, the difference in energy between the higher and lower energy shells produces an X-ray.

This represents the emission of X-rays. The energy of electrons which gets transferred depends on which shell it gets transferred from. Energy-dispersive spectrometer measurements are made of the quantity sample. The distinct X-ray’s energy and the variation in energies between the two lobes that are produced can be used to identify an entity’s component constitution. The qualitative composition of the sample can be obtained from the position of the peak [90].

### 2.6. Magnetometer for Vibrating Samples

The attraction of a sample may be measured using vibrating sample magnetometry (VSM). A pair of pick-up coils and the synthesized material are made to vibrate simultaneously. The coils are subjected to a perpendicular direct current (DC) magnetic field, which causes the sample to be magnetic. Somewhere in an instant, the oscillating magnetism creates permeability that fluctuates with time, thus, creating an alternating current gradient in the detecting coils. A lock amplifier detects the signal from the coils due to its extremely high gain and narrow bandwidth for a particular frequency [91]. The attractive instant of a material may be linked with its DC component that is obtained from the lock-in amplifier, and the magnetic field can be judged. This is the working principle of VSM.

A schematic view of the vibrating sample magnetometer is shown in the figure. From oscillating a material close to the receiver circuit, both the sample readings and electromotive force are measured concurrently. The system uses a solid gradiometer receiver circuit with variations in magnetization below 10^−6^ emu, at 1 Hz. The automation and control are carried out by using the MultiVu software application. The VSM motor modules are controlled by an optical nonlinear sensor output which is fully read from the VSM linear motor to set the oscillating location and intensity. The induced voltage is amplified in the pickup coil. The VSM-detecting module detects the lock-in range for the magnetization. This VSM detector uses the location sensor output as a standard for synchronized identification. Basic encoding data from the VSM nonlinear sensors are then utilized to decode the input data sent by the VSM motor modules. With an inbuilt modulated signal aspect, impulses from the decoder and an enhanced signal from the picking circuit are detected by the VSM sensor. Such impulses are once again delivered to the device’s VSM software through the CAN bus, and the magnetization curves are produced [92,93,94].

## 3. Results and Discussion

### 3.1. X-ray Diffraction Analysis

**Figure 1:**
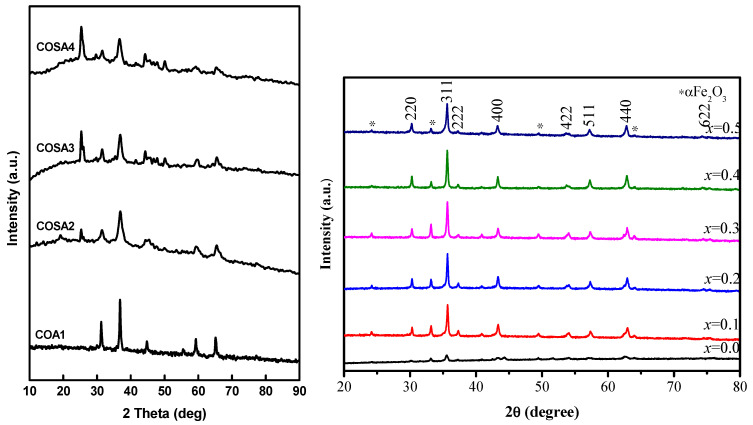
XRD mapping of Sr-doped CoAl_2_O_4_ nanoparticles.

The isotopic ratios of copper aluminosilicate nanopowders are observed under X-ray dispersion. It is observed that the entire diffraction pattern indicates a good crystalline structure. These Sr-modified chrome aluminosilicate reflection levels (220), (311), (400), (422), (511) and (440) are referred for diffraction peaks of 31.21°, 36.75°, 44.79°, 55.53°, 59.28° and 65.18°, correspondingly. Their spinel ferrite form having Fd-3m spacing class is shown by all the scattering spikes matching closely to the EDX analysis (JCPDS card number 82-2239) [95]. SrO nanomaterials possess contaminant spikes at two quantities of 26.51, which is in excellent accord with the Sr-doped CoAl_2_O_4_ systems (Figure 1). Utilizing (311) reflecting planes, its mean value for the shape factor is computed, employing Scherrer–Debye’s Equation (2).

(2) $$L=\frac{0.89\lambda }{\beta \cos\theta }$$

where *L* is indeed the shape factor, depending on the reflection coefficient, and the effective area at twice maximal (FWHM) of both the measured absorption edge, considering the X-ray origin frequency of 0.15406 nm. This Sr-treated chrome aluminosilicate crystalline phase (311) is determined to have a mean particle height of 14 to 20 nm [96,97,98,99]. The lattice parameter of the Co_1−*x*_Sr*_x_*Al_2_O_4_ (0 ≤ *x* ≤ 0.5) nanoparticles is computed utilizing Equation (3), as follows:

(3) $$a=d_{hkl}\sqrt{\left(h^{2}+k^{2}+l^{2}\right)}$$

where *d_hkl_* is the interatomic width determined by lattice constants; the crystalline surfaces’ *h*, *k*, and *l*; and the structural factor. Its monoclinic crystal is tasked with designing the crystallite size “*a*”, which is found between 8.111 Å and 8.122 Å. The estimated crystal size “*a*” agrees well with the number that was originally disclosed (8.106 Å) [100].

### 3.2. Evaluation Using a High-Resolution Electron Microscope (HR-SEM)

**Figure 2:**
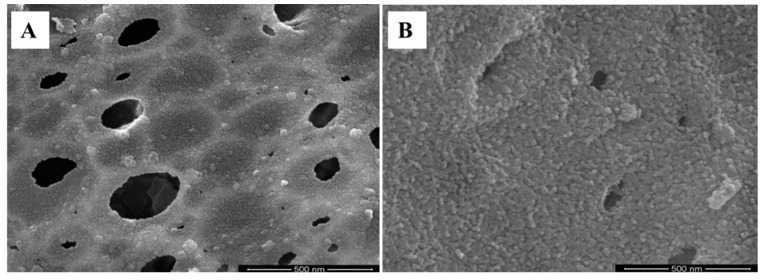
HR-SEM analysis of Sr-doped CoAl_2_O_4_ nanoparticles, (**A**) coalescence agglomeration of nanoparticles and (**B**) aggregation of nanoparticles.

**Figure 3:**
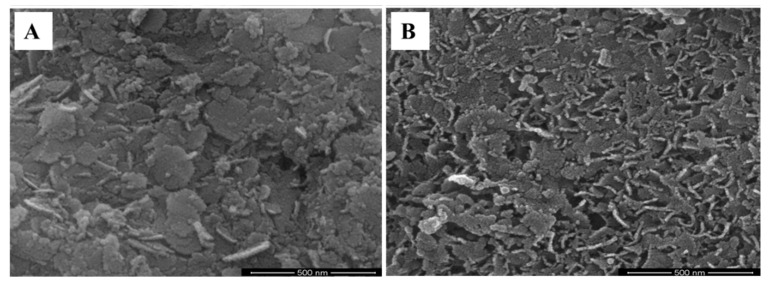
HR-SEM analysis of Sr-doped CoAl_2_O_4_ nanoparticles. (**A**) Porous and (**B**) spherical morphology.

The external geomorphology of Sr-doped CoAl_2_O_4_ nanoparticles is analyzed by high-level motion-testing electron microscopy. The HR-SEM descriptions observed cobalt aluminate nanoparticles, whose notable tendencies of agglomerated coalescence, and aggregation are seen in Figure 2A and 2B respectively, while the porous, and spherical morphology are shown in Figure 3A and 3B, respectively. The bulbous-aggregated shape is seen in Co_1−*x*_Sr*_x_*Al_2_O_4_ (0 ≤ *x* ≤ 0.5) nanoparticles, which is due to a lower-energy release in the combustion procedure [101,102,103,104].

### 3.3. Elemental Examination Using Energy-Dispersive X-ray Spectroscopy

**Figure 4:**
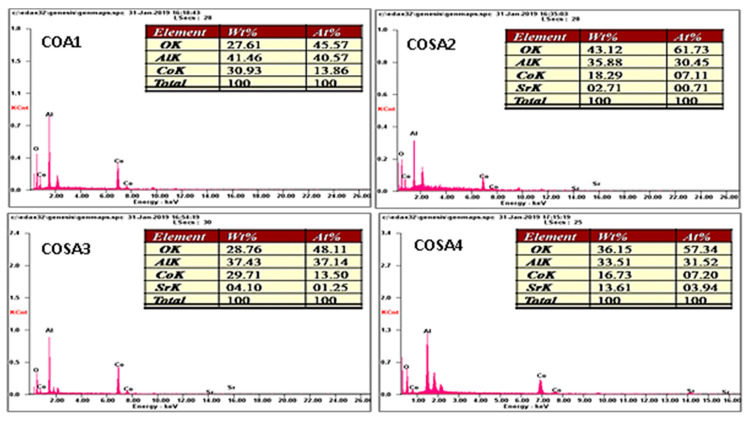
EDX spectra of Sr-doped CoAl_2_O_4_ nanoparticles.

The energy-dispersive X-ray spectroscopy based on the Photon flanking X-ray examination of Co_1−*x*_Sr*_x_*Al_2_O_4_ (0 ≤ *x* ≤ 0.5) nanomaterials revealed their chemical compositions, and are accordingly depicted in Figure 4. The elemental presence of Co, Sr, Al, and O, show excellent chrome aluminosilicate particulate replacement [105,106,107,108].

### 3.4. UV-Visible Diffused Reflectance Spectroscopy (DRS) Analysis

Energy-dispersive spectrometry in the ultraviolet range was verified for Sr-doped CoAl_2_O_4_ nanoparticles and was used to verify the energy band gap. The energy band gap value was calculated by the Taut relation [109,110,111]. Generally, the Kubelka–Munk function *F*(*R*) was used to convert the diffused reflectance into the absorption co-efficient, as shown in Equation (4).

(4) $$\alpha =F(R)=\frac{{\left(1-R\right)}^{2}}{2R}$$

If *R* represents reflectivity, *F*(*R*) seems to be the Kubelka–Munk function, while *A(hν − E_g_)^n^* seems to be the transmittance. Consequently, the equation’s relationship is given in Equation (5).

**Figure 5:**
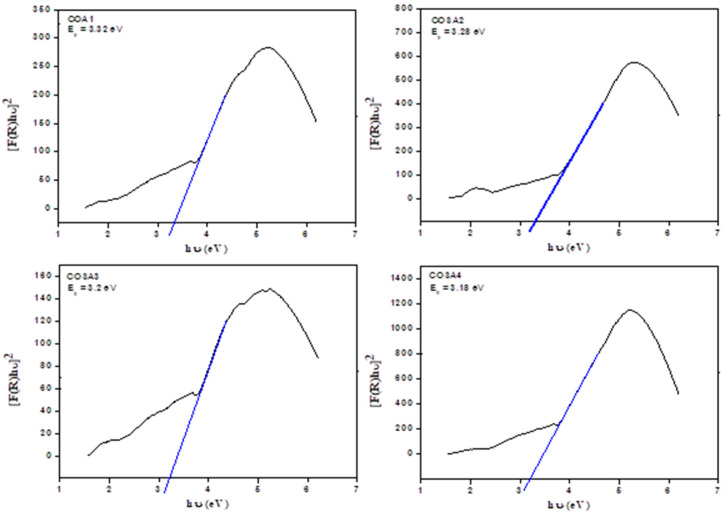
(*F*(*R*)*hν*)^2^ versus the band gap of Co_1−*x*_Sr*_x_*Al_2_O_4_ (0 ≤ *x* ≤ 0.5) nanoparticles.

*F*(*R*)*hν* = *A*(*hν – E_g_*)*^n^* (5)

where *n* = 2 and 1/2 represent the allowed direct and indirect transitions, thereby giving direct and indirect band gaps, respectively. A Tauc plot, between (*F(R)hν*)*^2^* beside the band gap (*hν*) for the cobalt aluminate nanoparticles, is shown in Figure 5, which represents the extrapolation of linear positions in the plots to (*F(R)hν*)*^2^* = 0 and gives the estimated direct band gap values [112]. The calculated direct band gap values of Sr-doped cobalt aluminate nanoparticles are found to be 3.32 eV, 3.28 eV, 3.2 eV, and 3.18 eV, respectively [113]. The black line represent the bandgap line for COAS1, COAS2, COAS3 and COAS4 compounds respectively, while the blue line in the graph represents the fitted line. 

### 3.5. FT-IR Spectra

**Figure 6:**
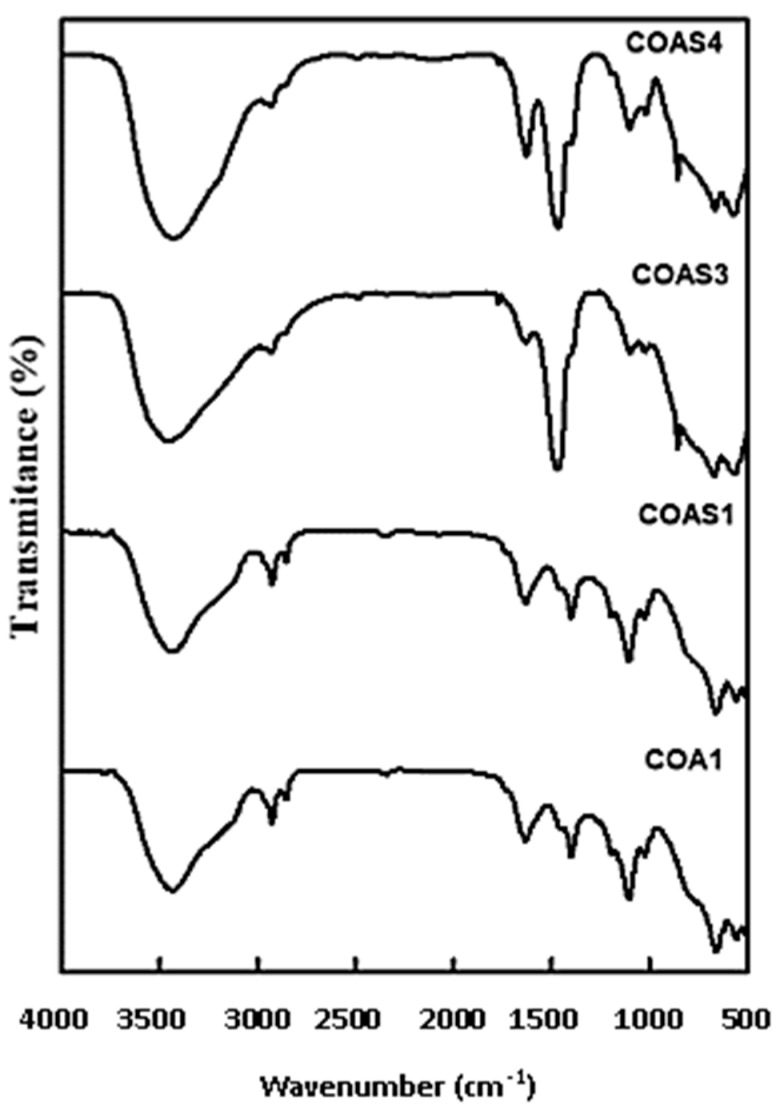
FT-IR spectra of Sr-doped CoAl_2_O_4_ nanoparticles.

Figure 6 shows the FT-IR band of Co_1−*x*_Sr*_x_*Al_2_O_4_ (0 ≤ *x* ≤ 0.5) nanoparticles made by the combustion method. At high space temperatures, the FT-IR range remained in the 4000–400 cm^−1^ scale. The wide range at 3439 cm^−1^ is due to the surface-absorbed water molecules’ -OH-reaching tremors [114]. Its C-H force is strong and is related to the group between 2920 and 2847 cm^−1^. Its distinctive wavenumber at 1631 cm^−1^ and 1435 cm^−1^ are attributed here to the coupling of the amino and carboxyl groups due to the residues of ionic species (such as COO^−^) mostly on the carbon surface [115]. The ensembles among 560–1100 cm^−1^ are due to the spinel structure of Sr-doped CoAl_2_O_4_ nanoparticles, respectively [116].

### 3.6. Magnetization Analysis

**Figure 7:**
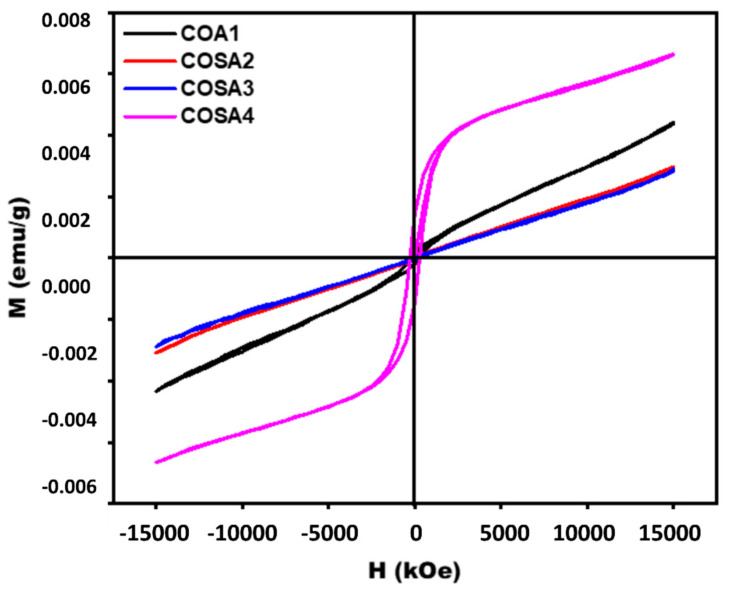
Magnetization curves of Sr doped CoAl_2_O_4_ nanoparticles.

The Sr-doped CoAl_2_O_4_ nanoparticle magnetization was examined at 300 K with just an applied field, which ranged from −15 kOe to +15 kOe. Behavior plots of the magnetic induction (M) against the magnetic field (H) are shown in Figure 6. The hysteresis loop is used to calculate the values of remanence magnetization (Mr), coercivity (Hc), and saturation of the magnetic induction (Ms). Because the tetrahedral and octahedral sites are filled by the divalent (Co^2+^) and trivalent (Al^3+^) metal ions, cobalt aluminate nanoparticles have such a ferromagnetic nature and a characteristic spinel structure. The high anisotropy, cationic redistribution, and exterior defect of cobalt aluminate nanoparticles are found to be responsible for the coercivity values of pure and Sr-doped cobalt aluminate (270.59 Oe) [117]. As shown in Figure 7, both Mr and Ms values are 5.08 emu/g and 20.54 emu/g, respectively. These values depend on the cobalt aluminate’s size, or the size and shape of the crystallites [118].

## 4. Conclusions

The characterization of cobalt aluminate nanoparticles for structural morphology and optical properties has gained significance due to their applications as pigment layers in luminescent materials. However, the use of a suitable fuel material for the combustion process is a big challenge that has been addressed in the present work. Co_1−*x*_Sr*_x_*Al_2_O_4_ (0 ≤ *x* ≤ 0.5) L-alanine, in the present work, was used as a fuel for the combustion process that produced nanoparticles. The cubic spinel with the Fd-3m space group was confirmed by the XRD. The lattice parameter value ranged from 8.112 Å to 8.122 Å. Whereas the EDX analysis demonstrated the presence of Co, Sr, Al, and O, the HR-SEM analysis revealed the surface morphology of the nanoparticles. The actual energy gap varied from 3.32 eV to 3.18 eV based on the absorption spectra. All the functional groups of Sr-doped CoAl_2_O_4_ spinel nanoparticles were detectable using FT-IR. The hysteresis analysis produced the Mr and Ms values (remanence ratio), which were found to be 5.08 emu/g and 20.54 emu/g, respectively. These values depend on the size and shape of the crystallites in the Sr-doped cobalt aluminate and are close to that of the magnetization value requirements for magnetic nanoparticles.

## Data Availability

Not applicable.
